# CoronaVac can induce the production of anti-SARS-CoV-2 IgA antibodies in human milk

**DOI:** 10.6061/clinics/2021/e3185

**Published:** 2021-06-23

**Authors:** Valdenise Martins Laurindo Tuma Calil, Patricia Palmeira, Yingying Zheng, Vera Lúcia Jornada Krebs, Werther Brunow de Carvalho, Magda Carneiro-Sampaio

**Affiliations:** I Instituto da Crianca e do Adolescente, Hospital das Clinicas HCFMUSP, Faculdade de Medicina, Universidade de Sao Paulo, Sao Paulo, SP, BR; II Laboratorio de Pediatria Clinica (LIM36), Departamento de Pediatria, Hospital das Clinicas HCFMUSP, Faculdade de Medicina, Universidade de Sao Paulo, Sao Paulo, SP, BR; III Departamento de Pediatria, Faculdade de Medicina FMUSP, Universidade de Sao Paulo, Sao Paulo, SP, BR

To the Editor,

Human milk is the external secretion with the highest immunoglobulin A (IgA) concentrations, mostly produced in the lamina propria of mammary glands by plasma cells (1). The milk antibody repertoire is quite similar to the one observed in the blood; however, the levels of antibodies against enteric and respiratory pathogens are usually higher in the colostrum and mature milk than in the serum. Maternal immunization can elicit systemic immunoglobulin G (IgG) and mucosal IgA, IgM, and IgG responses that confer protection to the newborn infants (2,3,4).

During the current pandemic, milk anti-SARS-CoV-2-specific IgA antibodies have been found in 23.1% of 2,312 previously infected lactating women (5,6). In an Israeli prospective cohort, milk samples of 84 breastfeeding women were analyzed before immunization and then weekly for six weeks after immunization. All the mothers received two doses of the Pfizer-BioNTech vaccine 21 days apart (7). The levels of IgA antibodies were significantly elevated two weeks after the first dose, with 61.8% of the samples testing positive (86.1% at week 4—one week after the second dose, and 65.7% at week 6).

Here, we present data from an initial study on the presence of anti-SARS-CoV-2 IgA antibodies in human milk samples obtained from volunteers during the immunization process promoted by HC-FMUSP in January (17^th^-21^st^) and February (15^th^-18^th^), 2021. The preparation “CoronaVac” (an inactivated vaccine), produced by Sinovac Biotech Ltd. (China) and Instituto Butantan (Brazil), was administered to all healthy employees in two doses, four weeks apart. A total of 170 samples were collected. All the 20 milk donors were HC-FMUSP employees and were breastfeeding at the time of the first immunization phase and voluntarily donated 5-10 mL milk samples before the first dose and seven more samples weekly for three weeks after the second dose. Milk samples were collected four months after the first dose from 10 mothers to evaluate the persistence of SARS-CoV-2-specific IgA antibodies. Milk was collected by the donors themselves into sterile containers after careful local antisepsis with sterile water. Manual expression or milk pump were used for sample collection after rigorous handwashing. The milk was stored at home by the donor at -20^o^C until delivery to the laboratory (LIM-36-ICr).

The study was approved by the Institutional Ethics Board (CAAE: 45565121.2.0000.0068), and written informed consent was obtained from all the participants. The levels of IgA antibodies that specifically bind the S1 domain of the spike protein (including RBD-Receptor Binding Domain) were semiquantitatively analyzed using the Euroimmun anti-SARS-CoV-2 S1 ELISA kit. The results were presented as the ratio of the optical density of the samples and the optical density of the calibrator (both read at 450 nm, using a reference wavelength of 620 nm), and ratios above 0.8 were considered positive. One-way ANOVA followed by Tukey’s multiple comparison tests were used in the statistical analysis (GraphPad v.7.0 Software Inc., San Diego, CA, USA), and statistical significance was set at *p<0.05*.

No significant adverse reactions were reported in either the mothers or their babies. The mean maternal age was 35.6 (±3.2) years at the time of the first dose, with a mean nursing period of 11.2 (±8.7) months, quite similar to the Israeli study, which was 10.3 months (7).

Of the 20 mothers, 16 were COVID-negative at week 0 (Figure 1). Despite an increase in the mean levels of anti-SARS-CoV-2-specific IgA in the first two weeks after the first dose, significantly higher mean values were obtained only at weeks 5 and 6. Ten mothers presented specific IgA antibody levels above the seroconversion value at week 7 (21 days after the second dose). Among the ten mothers who donated a sample four months after the first dose, five still had specific IgA levels above the seroconversion value at that time**.** In our series, four mothers had COVID-19, of whom three presented high levels of anti-SARS-CoV-2 IgA antibodies in W0 (data not shown). One of them donated her milk four months after the first vaccine dose and still had high specific IgA levels (anti-SARS-CoV-2-specific IgA ratio=4.0).

This study strongly reinforces that mothers should continue breastfeeding their children after vaccination against SARS-CoV-2 and even after infection (5-7). As for other respiratory infections, maternal anti-SARS-CoV-2 immunization should protect infants with systemic IgG and milk IgA providing local mucosal defense, as demonstrated by Gray et al. (8) in a large group of pregnant and lactating women who received Pfizer-BioNTech vaccine where all cord blood and breastmilk samples presented specific IgG and IgA antibodies, respectively. Therefore, to analyze both the placental transfer of anti-SARS-CoV-2 IgG and production of IgA in early milk, we are planning an equivalent protocol with “CoronaVac” immunization during pregnancy involving the collection of maternal and cord blood, colostrum, and milk during the first two post-delivery months (3,4).

## AUTHOR CONTRIBUTIONS

Calil VMLT, Palmeira P, and Carneiro-Sampaio M contributed substantially to the study conception and design, data analysis and interpretation, manuscript writing and editing. Zheng Y was responsible for sample collection, laboratory, and statistical analyses. Krebs VLJ and Carvalho WB were responsible for revising the manuscript. All of the authors critically revised the manuscript and approved its final version.

## Figures and Tables

**Figure 1:**
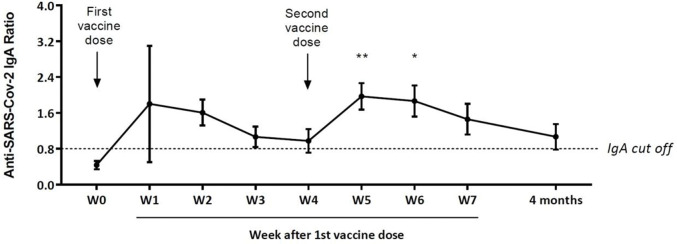
Anti-SARS-CoV-2-specific IgA ratios (mean±standard error) in milk samples collected over time (Weekly-W) from 16 healthy mothers previously COVID-negative after a 2-dose schedule of the CoronaVac vaccine (Sinovac Biotech Ltd., China). The last withdrawal was performed four months after the first dose in ten mothers. **p<0.01; *p<0.05.
